# Structure and elements of library evidence synthesis services: a content analysis of publicly available information

**DOI:** 10.5195/jmla.2026.2263

**Published:** 2026-07-01

**Authors:** Jaena Manson, Anna M. White, Emily Metcalf

**Affiliations:** 1 jaena.manson@rosalindfranklin.edu, Scholarly Communications Librarian, Boxer Library, Rosalind Franklin University of Medicine and Science, North Chicago, IL; 2 whitean2@gvsu.edu, Science & Health Sciences Liaison Librarian, University Libraries, Grand Valley State University, Grand Rapids, MI; 3 metcalem@gvsu.edu, Liaison Librarian – Research, Instruction, and Scholarly Communications, University Libraries, Grand Valley State University, Grand Rapids, MITN

**Keywords:** Evidence Synthesis, Library Services, Content Analysis

## Abstract

**Objective:**

Through a content analysis of public websites, this paper provides a centralized and summarized picture of evidence synthesis (ES) service characteristics at academic libraries and medical schools and centers in the United States in the mid-2020s.

**Methods:**

Our team identified 116 institutions classified in the 2021 Carnegie Classification as R1, R2, or Medical Schools and Centers that had an ES service. These services were coded for service characteristics. Using frequency and comparison tables, we answered the following research questions: What are the common structures and characteristics of ES services, within and independent of Carnegie Classification of Research Activity? Is there any relationship between Carnegie Classification of Research Activity and Size & Setting and characteristics of ES services?

**Results:**

We found that services often used tiers to delineate between services offered, with higher tiers requiring more responsibilities from the library or information professional. Some universities and medical schools limit librarian involvement with ES projects based on user role (student, faculty, researcher, etc.) or user affiliation with a college or department. Librarian acknowledgment or authorship are common in return for engagement in ES projects. Fees and Memoranda of Understanding were uncommon but do exist at some institutions.

**Conclusion:**

Librarians seeking to create or update their ES service can benefit from this ‘data snapshot,& as it will allow them to see the service elements under consideration by other institutions and offer ideas for their own services. Overall, we found that there were more similarities than differences between basic ES service structures (e.g. tier amounts/categories, presence of fees, etc.), independent of classification type or size setting. We recommend that librarians utilize this paper and the associated data in order to identify which institutional elements are important in their context for ideas in improving their ES service.

## INTRODUCTION

Evidence synthesis (ES) reviews have risen in popularity as a publication type in recent decades. Nearly 80 reviews were published per day in 2019, a 20-fold increase over the previous 20 years, a trend that continued through the 2020s [1, 2]. Studies suggest that many published ES reviews were of low quality, noting reporting and reproducibility as two areas of concern [3, 4, 5, 6, 7]. Librarians and information professionals are well-situated to participate in ES projects to increase the rigor of the creation, completion, and reporting of the search as well as to provide guidance on ES methodology, which research suggests produces higher-quality published work [4, 7, 8, 9].

Libraries have responded to this increase in ES projects, the need for improved review quality, and the evidence supporting the inclusion of information professionals in research teams in part by creating formal ES services within their libraries. Much of the literature regarding these services are case reports describing implementation of services at a particular institution within a specific context, and general advice for other institutions interested in setting up a service [10, 11, 12, 13, 14, 15, 16, 17, 18, 19]. These case reports are helpful in their detailed, if limited, perspectives, but we identified a need for a more systematic evaluation of the current landscape of ES service that provides information on common structures and patterns in practices to inform those creating new services. While many institutions have public webpages or LibGuides advertising ES services, busy librarians may not have the time or resources to investigate every existing case and model. In response, our project sought to provide a centralized, summarized picture of ES services at academic libraries and medical centers in the United States in the mid-2020s.

This project builds upon previous work in mapping ES services. Comer et al has provided one model for sharing service information in the OSF Cumulative Repository for Evidence Synthesis and/or Systematic Review Service Material [20]. This is a helpful repository; however, at the time of writing in 2025, the last update was marked June of 2023, and the information included is disparate rather than synthesized. Other projects have contributed findings on particular aspects of the ES service landscape: Westmark et al conducted a 2022 survey of 127 international health sciences institutions to examine patterns in librarian practice, including presentation of search methods, deduplication practices, fee-based models, and other practical elements of what they refer to as ‘librarian-mediated search services’ [21]. Similarly, Boden, Bolton, and Gerrard surveyed 94 Canadian librarians to identify common disciplinary areas, librarian titles, and librarian roles within ES work, in addition to describing common barriers to success [22]. Lee et al. analyzed systematic review online library guides for 18 English-speaking universities to determine whether they were primarily informational or educational in nature [23]. Our project joins this conversation in several notable ways: rather than collect data via survey, we have, like Lee et al., chosen to analyze public-facing information, which does not rely on response rate or the subjective responses of respondents, and increased the sample size]. The information on static library guides provides a snapshot of concrete, objective data to examine. Additionally, we examine the advertised structure of each service and expand upon the service structures and characteristics examined by Westmark to create a more comprehensive list of librarian duties within ES services using a deductive coding method. Finally, our project includes United States-based library ES services by institution classification, which provides a unique perspective on how trends and commonalities are distributed across a range of different types of institutions.

To this point, through a content analysis of library webpages, we sought to answer the following questions:

- What are the common structures and elements of ES services within and independent of Carnegie Classification of Research Activity?
- Are there any trends between Carnegie Classification of Research Activity and ES services?
- Are there any trends between Institutional Size & Setting and the structure and elements of ES services?

## METHODS

### Selection Criteria

We limited our content analysis to schools with 2021 Carnegie Classifications of Doctoral Universities - Very high research activity (R1), Doctoral Universities - High research activity (R2) and Four-Year Medical Schools & Centers (Medical). While institutions in other classifications may also operate ES services, these classifications indicated a high research output and, in the case of Four-Year Medical Schools & Centers, a high degree of relevance to typical ES topics. The Carnegie Classifications are a “system for organizing the diverse set of degree-granting colleges and universities in the United States” [24]. A new edition of the Carnegie Classifications was released in 2025, after the completion of our data analysis. However, this reclassification would have introduced minimal changes to our dataset, and we chose to continue with the 2021 Classifications.

For the purposes of this project, an ES service is an advertised library service that is explicitly dedicated to assistance for ES reviews. We define ES reviews as any research in which literature is systematically searched for, reviewed, and synthesized using a reproducible method, including but not limited to systematic reviews, scoping reviews, umbrella reviews, and meta-analyses.ES reviews include systematic reviews and scoping reviews.

### Data Collection

Three hundred and seventeen institutions met the criteria for 2021 Carnegie Classification R1, R2, or Medical Schools and Centers. The set of 317 institutions was arranged alphabetically in Google Sheets, then split into thirds. Each reviewer was assigned 2/3 of the set, so that two reviewers were responsible for each third. Each reviewer then searched for a relevant review webpage for their 2/3 (211 of 317) of the institutions and identified whether that institution had an ES service according to our definition, resulting in two determinations for each institution. Each reviewer then resolved conflicting determinations for their remaining 1/3 of the institutions. For example, if Author 1 indicated that a service was present and Author 3 determined that there was no service, Author 2 made a final determination to resolve the conflict. This method allowed for an evenly distributed data collection workload across the three authors.

Of the 317 schools investigated, we found ES service webpages for 37% (118 of 317). We used this preliminary data to create a codebook of service features, tier types, librarian responsibilities, and more (see Appendix A for entire codebook). In order to achieve intercoder agreement, we used the subjective assessment method, which consisted of all of us coding the same ten services randomly identified using a number generator. Then all three of us coded these services and compared codes, discussing and resolving differences. This process allowed us to refine codes and develop a shared understanding of codes [25].

In the next round of data collection, we sought to identify the characteristics of each previously identified service. Again, each reviewer collected data for 2/3 (78 of 118) of the identified services. Each reviewer maintained individual data records in Google Sheets to reduce the potential for bias. Reviewers collected the following data from each service webpage, where it existed:

- Whether the service was fee-based and, if so, what is the fee structure
- Whether the service provided levels of support in tiers, and if so, how many
- Tier qualities such as librarian duties and what populations can utilize each tier
- Requirements for librarian/information professional authorship
- Supporting evidence for including information specialists in ES projects, such as guidelines and articles

The third reviewer settled any discrepancies among the first two reviewers.

### Tier Definition and Coding

Tiered service models are a common approach to differentiate level of librarian involvement in ES services on the basis of cost, activity, or time [13, 15, 26]. Tiers are particularly helpful for services experiencing a high project load, though they also help illustrate to new researchers how a librarian might be able to support their work and thus lower the barrier for those researchers to request assistance. We wanted to understand how services were differentiated through tiers, including the number and type of tiers as well as the duties expected of information professionals associated with tiers. Services were coded as untiered (one level of service), two tiers, or three or more tiers.

Through inductive coding, in which concepts and definitions arise from the data, we specified four tier types, characterized by the role of the librarian in an ES project: Instructor, in which a librarian’s role is primarily teaching about ES methods; Consultant, in which a librarian is providing feedback on an ES research project’s methodology or search strategy; Team Member, in which a librarian is heavily contributing to the ES research; and, when services advertised only one service level and did not define its service by librarian role, Untiered. These tiers are further defined in the Tier Characteristics section.

Services with more than one tier could have any combination of these categories, including two tiers of the same type, as compared to our definitions. For example, if a service lists two tiers, but both tiers describe librarian duties that we categorized as ‘Consultant’ tier types, we labeled both tiers ‘Consultant.’

When coding tier characteristics, such as librarian duties involved, tier availability, and acknowledgment expectations, we only coded characteristics that were unique to each tier at individual institutions. Tiered services are often cumulative: that is, each ‘higher’ tier includes all services provided at a ‘lower’ tier in addition to the ‘higher’ tier’s offerings. For the purposes of our investigation, we identified which duties were unique to each level at each institution to understand what differentiates each level from the others.

While coding the characteristics of tiers, we also sought to identify the librarian duties most frequently associated with authorship privileges. In some cases, these duties were listed on a service page in a separate table; in others, they were embedded into descriptions of each tier. We only coded duties that were explicitly and exclusively mentioned as necessary for authorship. For example, if “manages citation software” was listed as a behavior in a Consultant tier AND a Team Member tier, but authorship was only required at the Team Member tier, we did NOT code for it as an authorship duty because it was not unique to authorship. Our process allowed us to clearly define what duties are most frequently connected to librarians as authors instead of service providers.

### Data Analysis

We chose to use an inductive thematic analysis to identify and create characteristics, codes, and themes from the data. This approach allows the characteristics, codes, and themes to emerge from the data as our analysis progressed [27]. The strength of this method is descriptive consistency and the amount of data we were able to collect and may also provide a unique perspective into the ways that libraries market and describe an ES service.

We arranged our dataset of services alphabetically, then split it into thirds, assigning each reviewer 2/3. As a result, each service was initially coded by two reviewers. Authors 1 and 3 each reviewed the first third, authors 2 and 3 the second, and authors 1 and 2 the third. One-third of the data set was one service fewer, at 38 services rather than 39. The third reviewer resolved code discrepancies where the first two reviewers disagreed. After any discrepancies were resolved and the codes for each service were finalized, we analyzed the frequency of codes across services and Carnegie classifications to identify trends. We generated tables and charts in Excel and Google Sheets to organize and present the data, and further analysis tables are available in Appendix B.

## RESULTS

### Services Included for Analysis

Though we initially identified 118 services, 116 services were included in our data analysis (see Table 1 for school characteristics) because one institution had three locations with individual web presences referring to one service.

**Table 1:** Included Schools by Research Activity & Size & Setting Classification

Carnegie Classification - Research Activity	N of Schools Included in Final Analysis
R1	84
R2	25
Medical Schools & Centers	7
Total	116
Carnegie Classification - Size	N of Schools Included in Final Analysis
Very Small (<1,000 FTE)	1
Small (1,000-2,999 FTE)	5
Medium (3,000-9,999 FTE)	10
Large (≥10,000 FTE)	100
Total	116

### Fees

For the most part, services did not charge a fee to researchers (91%, n=105). Of those that did, 55% (6 of 11) charged fees if the systematic review was part of a funded research project. Likewise, 55% (6 of 11) of the services that charged a fee based the fee on tier, 55% (6 of 11) charged for amount of time spent on a project (55%, n=6 of 11), 18% (2 of 11) charged a standard fee, and 9% (1 of 11) charged for services based on how many databases were searched. Of the institution classifications, R1 schools (9 of 116) most frequently required fees. By size, large schools (8 of 116) most frequently required fees.

### Rationale for Librarian Participation/Authorship

50% of the services examined (58 of 116) provided some form of justification for including an information professional in their review process. Of these, 43% (25 of 58) provided more than one piece of evidence. The most common justification provided was the authorship recommendations put forth by the International Committee of Medical Journal Editors (ICMJE) [28], with 50% (29 of 58) of services citing this resource. 38% (22 of 58) referenced the Institute of Medicine [29], 21% (12 of 58) referenced the Cochrane Handbook for Systematic Reviews of Interventions [30], and 5% (3 of 58) referenced the Journal of the American Medical Association (JAMA) [31]. 43% (25 of 58) of services that provided a justification cited a different resource, most often these were published journal articles that provided evidence of higher quality research coming from information professional involvement in the search development process.

### Tier Characteristics

**Figure 1:**
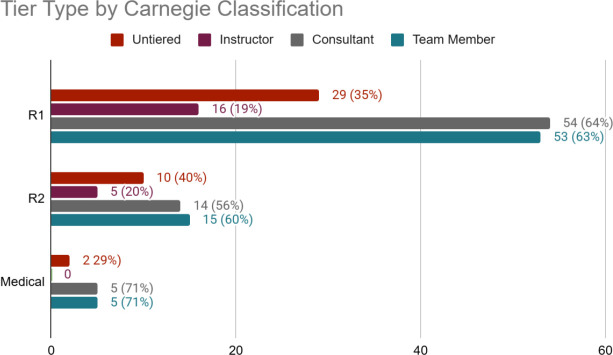
Tier Type by Carnegie Classification

We found that, across institution classifications and institution sizes, Consultant and Team Member tiers were the most common (see Figure 1).

In the following discussion, note that we are referring to unique duties to each tier at each institution rather than all the possible duties that a participating librarian might offer. See Table 2 for the top five duties in each tier category. For top five duties broken down by Carnegie classification, see Appendix B.

**Table 2:** Top 5 Duties (by Percentage) by Tier

Tier Level (N of schools with this level)	Included Duties (N of schools that list this duty)
Instructor (21)	90% (n=19) provide basic instruction on ES, includes identification of review type 43% (n=9) develop or revise initial search strategies 43% (n=9) recommend database/search environments 38% (n=8) instruct team on use of citation/screen software 10% (n=2) assist in research question development
Consultant (73)	79% (n=58) develop and/or revise initial search strategies 71% (n=52) recommend database/search environments 64% (n=47) provide basic instruction 63% (n=46) instruct team on citation/screening software 41% (n=30) assist in question/PICO development
Team Member (73)	96% (n=70) write final search strategy and database translations 95% (n=69) write search methods 89% (n=65) execute search and export results 53% (n=39) manage citation/screening software 52% (n=38) deduplicate results
Untiered (41)	56% (n=23) recommend databases/search environment 56% (n=23) develop or revise initial strategies 51% (n=21) provide basic instruction, including on review types 46% (n=19) instruct on citation/screening software 41% (n=17) assist in question development 41% (n=17) write final search strategy and translations 41% (n=17) write methods

### Instructor Tier

#### Prevalence

The Instructor tiers of services are those in which the librarian serves the ES project/team in a primarily educational capacity. In an Instructor tier, the librarian might provide class sessions, workshops, or group meetings focused on understanding the ES process and tools, but they do not participate in the review itself. This tier was least represented in our data; 18% (21 of 116) of services advertised an Instructor tier.

#### Duties

Ninety percent (19 of 21) of the services that listed an Instructor tier mentioned that librarians in this tier provide basic instruction on ES work, such as education on the distinction between types of ES projects. Forty-three percent (9 of 21) of the services stated that they recommend specific databases to research teams and provide feedback on initial search strategies, including guidance on query translation between databases. Thirty-eight percent (8 of 21) of services instruct researchers on the use of citation management or result screening software. Ten percent (2 of 21) guide researchers in the development of research questions appropriate for ES projects as part of Instructor duties.

### Consultant Tier

#### Prevalence

The Consultant tier are those in which the librarian provides targeted assistance to a particular ES team, typically on a time-limited basis at specific points in the project cycle. Librarians may review sections of work completed by the team and provide feedback and/or manage certain steps or tools, but are not typically official team members. Sixty-three percent (73 of 116) of services advertised a Consultant tier, and of the four tier types, Consultant was tied with Team Member for most represented tier type.

#### Duties

Within the Consultant tier the most common duty listed was developing or revising search strategies, with 79% (58 of 73) listing this duty. Seventy-one percent (52 of 73) recommend databases, search environments, and gray literature for projects. Sixty-four percent (47 of 73) instruct on evidence synthesis, including guidance on appropriate review type. Sixty-three percent (46 of 73) instruct on citation and screening software. Forty-one percent (30 of 73) assist with developing a research question within the duties of their Consultant tier. There are differences in how services grouped similar responsibilities. For example, while Instructor is a separate tier for some services, others bundle instruction duties into a Consultant tier when other tasks (such as the search strategy development) are required.

### Team Member Tier

#### Prevalence

**Figure 2:**
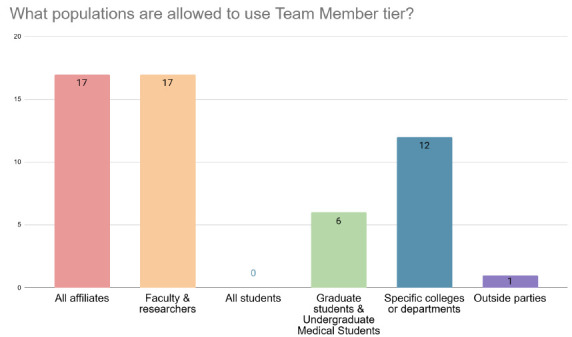
Specified included populations in Team Member tiers

The Team Member tier is defined as those in which the librarian serves as a full participant on a particular ES project. In this tier, librarians may be fully or partially responsible for completing certain aspects of the review. These responsibilities are sometimes in addition to consulting activities described above. Sixty-three percent (73 of 116) of services had a Team Member tier. See Figure 2 for what populations could utilize the Team Member tier (the numbers in table 3 are reflective of 67 institutions that explicitly mentioned population and are not mutually exclusive).

**Table 3:** Unique Duties of Authorship in Order of Prevalence (Top 5 Percentages)

Duties	N of Services (out of 34)
Writes search methods	79% (n=27)
Writes search strategy	76% (n=26)
Executes search & exports results	74% (n=25)
Manages citation/screening software	44% (n=15)
Deduplicates results	44% (n=15)
Completes flow diagram	38% (n=13)
Reviews manuscript	38% (n=13)

#### Duties

The most common duties of Team Member tiers were writing the final search strategy and translating the query for any identified databases, with these duties being mentioned by 96% (70 of 73) of services. Eighty-nine percent (65 of 73) specified that librarians execute the search and export results for the research team. Ninety-five percent (69 of 73) include authorship of the search methodology section of any manuscripts resulting from an ES project. Fifty-three percent (39 of 73) manage citation and screening software for the research team. Other frequently mentioned tier characteristics included deduplicating search results, which appeared in 52% (38 of 73) of services, and completing a PRISMA flow diagram, which appeared in 51% (37 or 73) of services.

### Untiered

#### Prevalence

Services coded as Untiered had only one service level and did not clearly define the role of the librarian within an ES project. Untiered services frequently listed a variety of potential tasks that a librarian may engage with throughout the ES process, often overlapping with typical Instructor, Consultant, and Team Member responsibilities. Thirty-five percent (41 of 116) of services were Untiered.

#### Duties

In Untiered services, the most common duties were developing or revising an initial search strategy for researchers and recommending databases: 56% (23 of 41) offered these services. Fifty-one percent (21 of 41) of Untiered services provide basic instruction on ES projects and 46% (19 of 41) provide instruction on citation or screening software. Forty-one percent (17 of 41) assist in question development, develop a final search strategy, and write the methods section of manuscripts or research projects. Untiered services reported the same most common tasks as the other three categories (providing basic ES instruction, developing search strategies, and writing methods sections). Ten percent (4 of 41) of Untiered services did not specify services offered.

### Service Requirements for Authorship

#### Authorship Responsibilities

Seventy-one percent (82 of 116) of services either required authorship or would negotiate authorship in one or more of their tiers. Out of those 82 schools that either required or negotiated authorship, only 41% (34 of 82) defined authorship duties. The most common duties that either required or called for negotiation of authorship were writing search methods and search strategy, as well as executing and exporting search results, deduplicating results, completing the flow diagram and reviewing the final manuscript before publication (see Table 3 & 4).

**Table 4:** Authorship/Acknowledgement Requirements by Tier

Tier	Authorship/Acknowledgement
Untiered (n=44)	23% (n=10) did not address acknowledgement 14% (n=6) authorship required 14% (n=6) authorship negotiable 5% (n=2) acknowledgment negotiable 2% (n=1) acknowledgement required
Instructor (n=21)	76% (n=16) did not address acknowledgement 14% n=(3) acknowledgement negotiable
Consultant (n=79)	38% (n=30) did not address acknowledgement 34%(n=27) acknowledgment required 11% (n=9) acknowledgment negotiable 3% (n=2) authorship negotiable
Team Member (n=74)	89% (n=66) authorship required 4% (n=3) did not address acknowledgment 4% (n=3) authorship negotiable 3% (n=2) acknowledgement negotiable 1% (n=1) acknowledgment required

#### Instructor

Fourteen percent (3 of 21) of universities with an Instructor tier either required or recommended a librarian acknowledgment or authorship credit. All three services stated that acknowledgment was negotiable.

#### Consultant

Fifty-two percent (38 of 73) of the Consultant tiers discussed acknowledgment or authorship. Of these, acknowledgment was required in over one third of the services. Authorship was never explicitly required at the Consultant tier, but was negotiable in 3% (2 of 73) of analyzed services.

#### Team Member

Ninety percent (66 of 73) of the services required authorship on any scholarly output resulting from the research. Four percent (3 of 73) listed authorship as negotiable, 3% (2 of 73) said acknowledgment was negotiable and 1% (1 of 73) of services stated that they required acknowledgment for engagement at this level.

#### Untiered

Fifteen percent (6 of 41) of Untiered services required authorship and a further 15% (6 of 41) stated authorship was negotiable; this split is understandable, given the wide range of librarian tasks represented in an Untiered service. Five percent (2 of 41) stated acknowledgement was negotiable and 2% (1 of 41) stated acknowledgement was required

#### Populations Served

Fifty-eight percent (67 of 116) of services make any mention of which populations can access their services and at which tier level. Our data reflects only those services that explicitly mentioned which populations were served. No services with a Team Member level listed “All Students” as a population that could access that level of librarian collaboration (see Appendix B).

#### Authorship by Institution Type

We found that a majority of services (71 of 116) required or negotiated authorship in some part of their service, independent of the Carnegie Classification or Size Setting. Services at R1 institutions reported the fewest authorship stipulations (69%, or 58 of 84 as compared to 72%, or 18 of 25 of R2 institutions and 86%, or 6 of 7 Medical Schools and Centers). However, there were a greater number of R1 institutions overall, representing a larger portion of our sample regardless of the lower percentage.

#### Memoranda of Understanding (MOU)

We found that some services chose to implement more formal understandings of librarian participation, such as Memoranda of Understanding (MOUs). MOUs can help review teams voice mutually agreed upon expectations and can introduce research teams to the types of tasks librarians may complete during a review. Twelve percent (14 of 116) of services mentioned an MOU. Ninety-three percent (13 of 14) of those services were at Large (over 10,000 FTE) institutions.

## DISCUSSION

Initially, we sought to answer the following questions:

- What are the common structures and elements of ES services within and independent of Carnegie Classification of Research Activity, as related to fees, service populations, and authorship?
- Are there any trends between Carnegie Classification of Research Activity and ES services?
- Are there any trends between Institutional Size & Setting and the structure and elements of ES services?

Our project revealed findings about the similarities between ES services at different Carnegie Classifications, the presence of fees, what populations can use ES services, how libraries communicate about their ES service and how involved librarians are in the ES process.

### Classification Similarities

There are more similarities than differences across Carnegie classifications with regards to structure and elements of ES Services. This may suggest that the ES process benefits from similar support from information professionals regardless of institution research output. These similarities could indicate that new services model their offerings on existing services; for instance, it was not uncommon for one Evidence Synthesis LibGuide to report that it was based on or created with support from another. In some ways, this is less surprising, as we began on our project with the assumption that new services would utilize existing resources to structure and market their own work. The consistency in elements across services may imply that service creators do not, and perhaps have no need to, limit themselves to peer institutions when searching for helpful models and language.

### Fees

The vast majority of services we explored did not seek monetary compensation for their services; even fewer required payment when the project was not grant-funded. This suggests that most libraries view their ES services as an expansion of normal library operations (in which institutional affiliates typically receive services at no additional charge or as a bundled payment for university services) rather than an extra or add-on offering. If demand for ES increases, ES services may wish to explore a fee-based model for reasons such as to compensate librarians for their labor, interlibrary loan costs, specialized software, or additional personnel. Whether a fee is necessary may be driven by the number of projects requiring support, which is influenced by the population that uses the service. We did not examine the correlation between librarian authorship and fee-based services or attempt to determine if librarians at fee-based services were faculty with scholarship responsibilities. A faculty librarian who serves as an author on an ES project may feel that the publication is ‘payment’ enough.

### Populations Served

ES projects assigned to students as part of degree completion are a subject of much discussion among librarians given the associated time and labor necessary to produce a quality product [32]. Librarians may provide basic instruction to undergraduates and graduate students working on these assignments; other services choose to support only those projects meant to produce publication-quality outputs authored by faculty and researchers. This may reflect the attitude at some institutions that instruction, even on the ES process, is within the scope of general instructional efforts rather than an ES service. It may be that ES instruction is taking place at more than 18% of the institutions selected, but is not reflected in the service itself. Even within a service’s designated population there are restrictions: some ES services will not work on projects authored by professional associations even if the primary investigator is an institutional affiliate. Librarians creating an ES service should consider if they will restrict support to particular institution groups, especially if there are many student projects in addition to research teams preparing for ES publication.

### Communication of Services

There may be a discrepancy between how ES services are marketed and how they operate in practice. For instance, though many services did not describe requirements or negotiations for authorship or acknowledgment of librarians, these conversations with research teams could be occurring on a case-by-case basis. Clarity in these expectations benefits both users of that service and creators of future services. Teams may increase the clarity of their service details by ensuring service information is centralized in one web location.

One common structural trend we found in ES services was a high number of Untiered services. These described a wide range of librarian tasks that overlapped with typical duties for other tiers. While we found defined tiers helpful in our analysis, we also acknowledge that there are benefits to this approach, with a less-defined librarian role or less-detailed public-facing service page. A more flexible service description allows for customization by the ES service with consideration to time commitments, complexity of project, funding, interpersonal relationships, and other variables that factor into participation. We find that a phrase such as “This page is a description of how a librarian may engage with your ES project. Actual participation will be discussed with your research team at the discretion of the librarian,” allows for clarity of expectation by the research team while preserving the right of the ES service to make changes.

### Librarians as Partners in Evidence Synthesis Research

Librarian involvement increases the quality of published ES projects [4, 7, 8, 9]. Half of services analyzed listed some kind of rationale for librarian involvement and/or authorship. This may suggest that librarians involved in these services have needed external support in negotiating partnership with research teams; authorship in particular is a sometimes-delicate discussion for researchers across disciplines, and librarians may value research and guidelines as they advocate for their own inclusion. As reported above, the ICMJE standards were the most frequently referenced rationale for librarian involvement in ES projects. It may be that services used the ICMJE authorship criteria, given the overlap between the standards and the most common responsibilities for authorship reported by services. For example, creating an appropriately rigorous search method is a ‘substantial contribution to the conception or design of the work,’ while writing manuscript search methods and/or completing the PRISMA flow diagram is ‘drafting the work.’

While some services had very clear guidelines and expectations for authorship or acknowledgement of librarian partners, many made no reference to crediting librarian involvement. We suspect that even though many services did not mention librarian authorship or acknowledgment, discussions regarding librarian authorship or acknowledgement are possibly occurring without being in the service’s public facing description.

As previously noted, institutions with larger populations or R1 status more often mentioned both MOUs and librarian authorship. It is possible that larger populations may mean more requests for ES support, which in turn may result in a need for clear agreements between research teams and librarians. Large institutions may also have more librarian support within services, which may make the creation and utilization of an MOU or authorship expectations more feasible than for smaller teams.

### Limitations

Our findings are limited by the fact that we only included R1, R2, and Medical institutions, and only US-based institutions. This means our findings can only be taken as an evaluation of those types of institutions and cannot be generalized to all ES services. In addition, we were limited by what information was available on public-facing websites. It is possible that some services were not marketed on public websites or that information on those websites were inaccurate or limited. Similarly, our data serve as a snapshot of a point in time of ES services.

Throughout our data collection and analysis process, services paused, began, re-structured, and made other changes that influence analysis. This is a fact of library services: they evolve to meet their institutional needs. For ease and clarity of data analysis, however, we chose an arbitrary data collection end point, and therefore our results best represent what ES services looked like in mid-to-late-2024. If we noticed that services had changed during data reconciliation (i.e. the website changed before a tie-breaker could resolve any coding discrepancies), we chose to include all codes entered at that point. This decision was reflective of our goal to map general trends in structures and elements of ES services rather than a perfect replica of each library’s service.

### Future research

More work can be done to map ES services more broadly through research projects that focus on areas that this project did not address, such as geographic locations outside of the U.S., other institutional classifications, and more. Follow-up work can be done by revisiting these institutional pages in the future to see if services have remained the same, or changed and in what ways. In addition, we intend to pursue qualitative research that builds upon this research by examining the experiences and perspectives of those creating and running these services beyond the already existing collection of case studies. This type of research would provide color and context to the trends we have identified in this project. In particular, qualitative research might look to identify trends and patterns in services that have long existed and evolved several times in comparison to newer or in-creation services, as well as the experiences of small ES teams (or “solo librarian” models) as compared to larger service models. Answers to these questions could further guide leaders and creators of ES services in their practice.

## CONCLUSION

There is an increase in ES projects as a form of scholarship across disciplines. Our project revealed the most common structures of ES services and what types of duties librarians contribute to ES reviews. We did not find that Carnegie Classification or Size and Setting had an impact on the structure and elements of ES services. To support these projects, it is possible that more libraries will begin services, and those that exist will adjust their offerings and marketing to meet demand. Librarians in both circumstances can benefit from this ‘data snapshot,’ as it will allow them to see the service elements under consideration by other institutions and offer ideas for their own services. In particular, librarians beginning services may avoid common pitfalls by viewing the patterns in established services. For example, librarians may begin their services already identifying authorship criteria rather than needing to retroactively define authorship for their research teams. We strive to make these patterns transparent and to reduce the workload of service creators and providers. Locating and analyzing over one hundred ES service webpages was a significant act of time and labor. We hope that those creating or evolving their own services can benefit from this synthesis without the need to duplicate our efforts.

## Data Availability

Data associated with this article are available in the Open Science Framework at https://osf.io/Q7J52/.

## SUPPLEMENTAL FILES

**Appendix A:** Codebook
