# Supplementary material for: Structure and elements of library evidence synthesis services: a content analysis of publicly available information
Source: J Med Libr Assoc. 2026 Jul 14;114(3):266–77. doi: 10.5195/jmla.2026.2263 (PMC13367309; doi:10.5195/jmla.2026.2263)
Supplement: Supplementary file 1 — Appendix A: Codebook [file jmla-114-3-266-s01.docx]

| **Code Category** | **Code** | **Definition** |
| --- | --- | --- |
| **Fees** | 1. Fee-based |  |
|  | 1. Fee-based if funded |  |
|  | 1. Fee-based for non-affiliates |  |
|  | 1. Fee by time |  |
|  | 1. Fee by tier |  |
|  | 1. Fee by database |  |
|  | 1. Fee lump sum |  |
| **Number of Tiers** | 1. Untiered |  |
|  | 1. 2 Tiers |  |
|  | 1. 3 or More Tiers |  |
| **Tier Types** | 1. Instructor | Teaches team or members of team about or how to complete any step of the evidence synthesis process. This can take place in a classroom, workshop, or meeting setting. |
|  | 1. Consultant | Reviews, revises, guides, or provides suggestions/recommendations on any step of the evidence synthesis process. May complete pre-work (searching for existing protocols/SR), but does not manage search, software, or participate in manuscript review. |
|  | 1. Team Member | Manages search and participates in manuscript writing or revision, in addition to any combination of other services. |
|  | 1. Other/Undefined |  |
| **Tier Characteristics** | 1. Provides basic instruction on evidence synthesis (process and methods) | Includes identification of review type |
|  | 1. Assist in question/PICO development |  |
|  | 1. Pre-search for existing systematic reviews/protocol |  |
|  | 1. Instructs team on protocol registration |  |
|  | 1. Participates in protocol creation |  |
|  | 1. Recommends databases/search environments | Includes Gray Literature |
|  | 1. Develops and/or revises initial search strategies | Can include education and advice on search translations |
|  | 1. Writes final search strategy and translations |  |
|  | 1. Executes search & exports results | Runs the search in all identified search environments, reruns any searches as necessary, exports results and shares with the researchers |
|  | 1. Deduplicates results |  |
|  | 1. Retrieves full-text articles | Can include educating on how to retrieve full-text articles |
|  | 1. Instructs team on use of citation/screening software |  |
|  | 1. Manages citation/screening software |  |
|  | 1. Completes flow diagram | Completed or assisted in completion |
|  | 1. Writes search methods |  |
|  | 1. Reviews manuscript |  |
|  | 1. Suggests journals for publication |  |
|  | 1. Services not specified |  |
|  | 1. All affiliates |  |
|  | 1. Faculty & researchers |  |
|  | 1. All students |  |
|  | 1. Graduate students and UGMS |  |
|  | 1. Specific college or department affiliates |  |
|  | 1. Outside parties |  |
|  | 1. MOU encouraged or required |  |
|  | 1. Authorship required |  |
|  | 1. Acknowledgement required |  |
|  | 1. Authorship negotiable |  |
|  | 1. Acknowledgement negotiable |  |
| **Authorship Responsibilities** | 1. Provides basic instruction on evidence synthesis (process and methods) |  |
|  | 1. Assist in question/PICO development |  |
|  | 1. Pre-search for existing systematic reviews/protocol |  |
|  | 1. Instructs team on protocol registration |  |
|  | 1. Participates in protocol creation |  |
|  | 1. Recommends databases |  |
|  | 1. Suggests revisions to search strategies |  |
|  | 1. Writes search strategy |  |
|  | 1. Executes search & exports results |  |
|  | 1. Deduplicates results |  |
|  | 1. Retrieves full-text articles |  |
|  | 1. Instructs team on use of citation/screening software 2. Manages citation/screening software |  |
|  | 1. Completes flow diagram |  |
|  | 1. Writes search methods |  |
|  | 1. Reviews manuscript |  |
|  | 1. Suggests journals for publication |  |
|  | 1. Services not specified |  |
| **Rationale for Authorship** | 1. International Committee of Medical Journal Editors (ICMJE) |  |
|  | 1. Institute of Medicine (IOM) |  |
|  | 1. Journal of the American Medical Association (JAMA) |  |
|  | 1. Cochrane |  |
|  | 1. JBI |  |
|  | 1. Other |  |
| **Where was the Information Located** | 1. LibGuide (or other Springshare item) |  |
|  | 1. Library webpage |  |
|  | 1. Non-library webpage |  |
