# Supplementary material for: Structure and elements of library evidence synthesis services: a content analysis of publicly available information
Source: J Med Libr Assoc. 2026 Jul 14;114(3):266–77. doi: 10.5195/jmla.2026.2263 (PMC13367309; doi:10.5195/jmla.2026.2263)
Supplement: Supplementary file 2 — Appendix B: Data Tables [file jmla-114-3-266-s02.docx]

Authors’ Note: None of these data are mutually exclusive (e.g. services often had more than one characteristic and the presence of one characteristic did not exclude the possibility of other characteristics noted).

*Presence of a Fee*

| **Service Fees** | **Number of Instances** |
| --- | --- |
| Fees for all projects or unspecified | 6 |
| Fee if project is funded | 6 |
| Fee for non-affiliates | 2 |

*Fee Structure*

| **Fee Calculation** | **Number of Instances** |
| --- | --- |
| By time | 6 |
| By tier | 6 |
| By database searched | 1 |
| Flat rate | 2 |

*Explicitly Included Populations by Tier Type*

| **Tier (N of schools with this level)** | **Populations available to** |
| --- | --- |
| Untiered (n=41) | 17% (n=7) available to faculty and researchers  15% (n=6) available to all affiliates  10% (n=4) available to specific colleges or departments  2% (n=1) available to graduate students and undergraduate medical students  2% (n=1) available to outside parties |
| Instructor (n=21) | 24% (n=5) available to a particular college or department  19% (n=4) available to all university affiliates  5% (n=1) available to faculty and researchers  5% (n=1) available to all students |
| Consultant (n=73) | 44% (n=32) available to all affiliates  14% (n=10) available to faculty and researchers  12% (n=9) available to a specific college or department  7% (n=5) available to graduate students and medical students  3% (n=2) available to all students  1% (n=1) available to outside parties |
| Team Member (n=73) | 23% (n=17) available to all affiliates  23% (n=17) available to faculty and researchers  16%(n=12) available to specific college or department  8% (n=6) available to graduate students and undergraduate medical student  3% (n=2) available to outside parties |


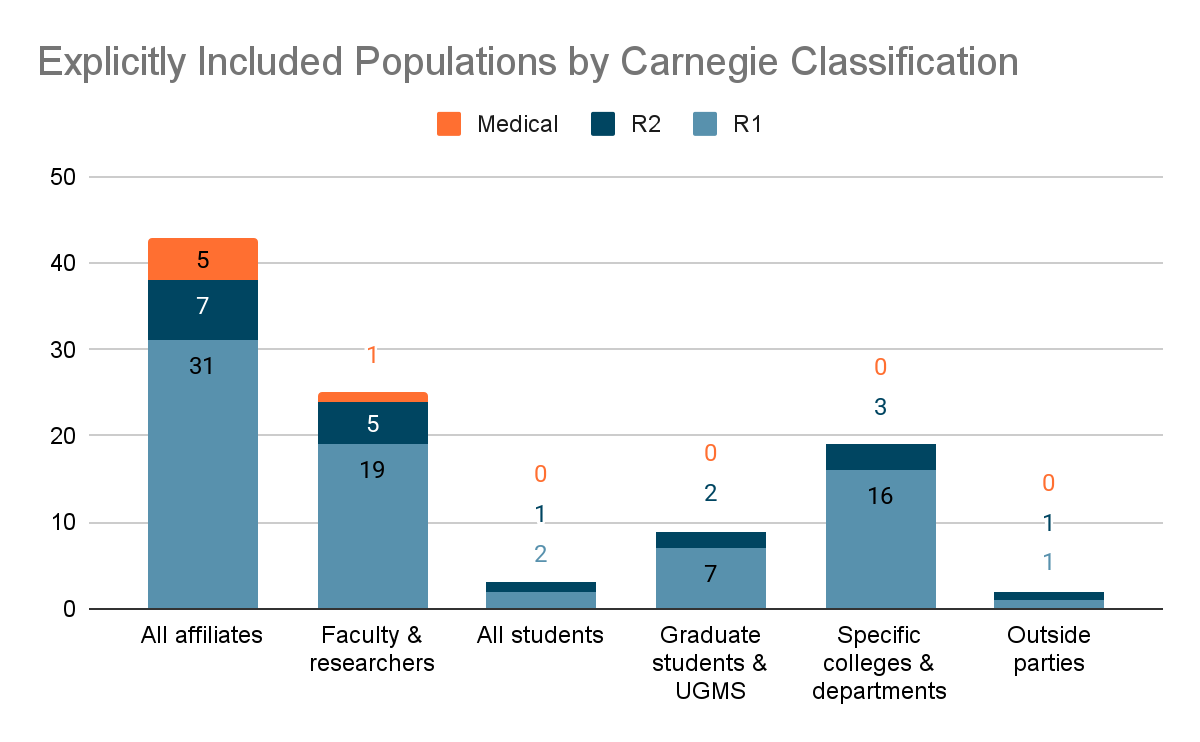


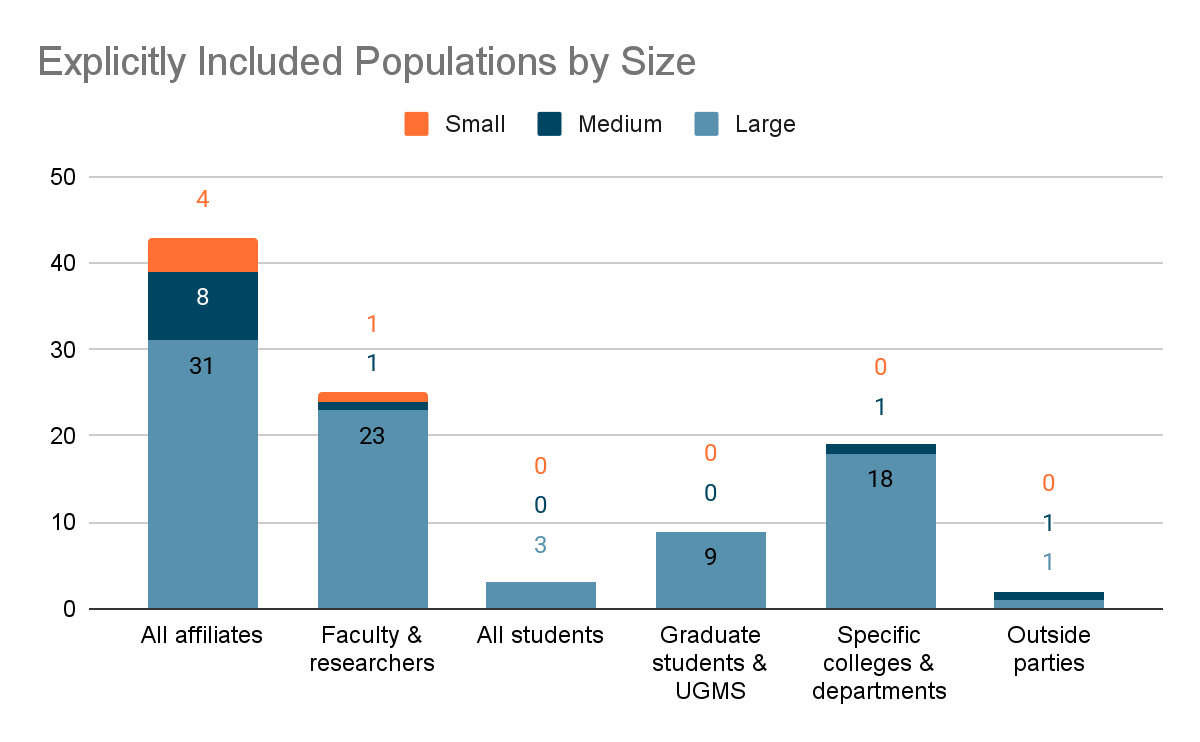


*Rationalization of Information Professional Involvement*

| **Rational Source** | **Number of Services** |
| --- | --- |
| International Committee of Medical Journal Editors (ICMJE) | 29 |
| Institute of Medicine (IOM) | 22 |
| Journal of the American Medical Association (JAMA) | 3 |
| Cochrane Handbook for Systematic Reviews | 12 |
| JBI Manual for Evidence Synthesis | 1 |
| Other | 25 |
| None | 58 |

Duties Associated with Authorship

| **Characteristic/Behavior** | **N of Services** |
| --- | --- |
| Writes search methods | 96% (n=27) |
| Writes search strategy | 93% (n=26) |
| Executes search & exports results | 89% (n=25) |
| Manages citation/screening software | 54% (n=15) |
| Deduplicates results | 54% (n=15) |
| Completes flow diagram | 46% (n=13) |
| Reviews manuscript | 46% (n=13) |
| Participates in protocol creation | 43% (n=12) |
| Recommends databases | 39% (n=11) |
| Retrieves full-text articles | 25% (n=7) |
| Assist in question/PICO development | 25% (n=7) |
| Pre-search for existing systematic reviews/protocol | 18% (n=5) |
| Provides basic instruction on evidence synthesis (process and methods) | 18% (n=5) |
| Suggests revisions to search strategies | 18% (n=5) |
| Instructs team on use of citation/screening software | 18% (n=5) |
| Instructs team on protocol registration | 14% (n=4) |
| Suggests journals for publication | 11% (n=3) |

*Mention of Population by Institution Type and Size Setting*

| **Carnegie Classification - Research Activity** | **N of Services that Mention Population** |
| --- | --- |
| R1 | 50 |
| R2 | 12 |
| Medical Schools & Centers | 5 |
| **Carnegie Classification - Size & Setting** | **N of Services that Mention Population** |
| Very Small (<1,000 FTE) | 0 |
| Small (1,000-2,999 FTE) | 4 |
| Medium (3,000-9,999 FTE) | 8 |
| Large (≥10,000 FTE) | 55 |
| **Total that Mention Population** | 67 |

*Presence of a Fee by Institution Type and Size Setting*

| **Institution Types** | **N have Presence of Fee** |
| --- | --- |
| R1 (84) | 9 |
| R2 (25) | 2 |
| Medical (7) | 0 |
| **Size of School** |  |
| Very Small | 0 |
| Small | 0 |
| Medium | 3 |
| Large | 8 |


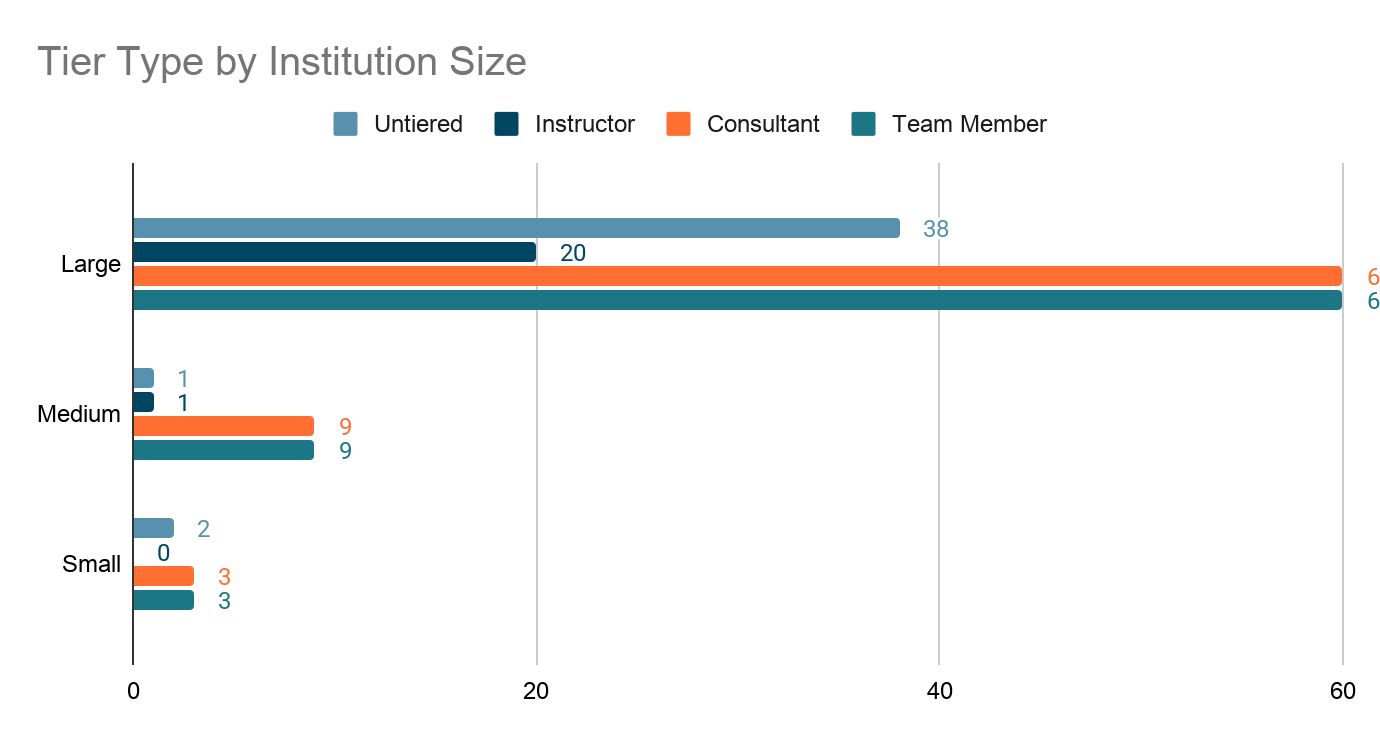


*Top 5 Duties (by Percentage) by Tier in R1 Schools*

| **Tier (N of R1 schools with this level)** | **Included Duties (N of schools that list this duty)** |
| --- | --- |
| Untiered (29) | 59% (n=17) Recommends databases/search environments  55% (n=16) Writes final search strategy and translations  55% (n=16) Provides basic instruction on evidence synthesis (process and methods)  52% (n=15) Develops and/or revises initial search strategies  48% (n=14) Assist in question/PICO development |
| Instructor (16) | 81% (n=13) Provides basic instruction on evidence synthesis (process and methods)  44% (n=7) Develops and/or revises initial search strategies  38% (n=6) Recommends databases/search environments  31% (n=5) Instructs team on use of citation/screening software  13% (n=2) Assist in question/PICO development |
| Consultant (54) | 80% (n=43) Develops and/or revises initial search strategies  70% (n=38) Recommends databases/search environments  65% (n=35) Instructs team on use of citation/screening software  63% (n=34) Provides basic instruction on evidence synthesis (process and methods)  37% (n=20) Assist in question/PICO development |
| Team Member (53) | 98% (n=52) Writes search methods  98% (n=52) Writes final search strategy and translations  94% (n=50) Executes search & exports results  74% (n=39) Manages citation/screening software  72% (n=38) Completes flow diagram |

*Top 5 Duties (by Percentage) by Tier in R2 Schools*

| **Tier (N of R2 schools with this level)** | **Included Duties (N of schools that list this duty)** |
| --- | --- |
| Untiered (10) | 70% (n=7) Develops and/or revises initial search strategies  60% (n=6) Recommends databases/search environments  60% (n=6) Instructs team on use of citation/screening software  50% (n=5) Writes final search strategy and translations  50% (n=5) Executes search & exports results |
| Instructor (5) | 100% (n=5) Provides basic instruction on evidence synthesis (process and methods)  60% (n=3) Recommends databases/search environments  60% (n=3) Instructs team on use of citation/screening software  40% (n=2) Develops and/or revises initial search strategies  20% (n=1) Instructs team on protocol registration  20% (n=1) Retrieves full-text articles |
| Consultant (14) | 71% (n=10) Develops and/or revises initial search strategies  64% (n=9) Recommends databases/search environments  57% (n=8) Provides basic instruction on evidence synthesis (process and methods)  50% (n=7) Assist in question/PICO development  43% (n=6) Instructs team on use of citation/screening software |
| Team Member (15) | 100% (n=15) Writes search methods  93% (n=14) Writes final search strategy and translations  67% (n=10) Executes search & exports results  47% (n=7) Deduplicates results  33% (n=5) Reviews manuscript  33% (n=5) Completes flow diagram  33% (n=5) Manages citation/screening software  33% (n=5) Instructs team on use of citation/screening software  33% (n=5) Recommends databases/search environments |

*Top 5 Duties (by Percentage) by Tier in Medical Schools*

| **Tier (N of R2 schools with this level)** | **Included Duties (N of schools that list this duty)** |
| --- | --- |
| Untiered (2) | *Not enough data. Only one of the two schools provided actual duties in this tier.* |
| Instructor (0) |  |
| Consultant (5) | 100% (n=5) Provides basic instruction on evidence synthesis (process and methods)  100% (n=5) Recommends databases/search environments  100% (n=5) Develops and/or revises initial search strategies  60% (n=3) Assist in question/PICO development  40% (n=2) Pre-search for existing systematic reviews/protocol  40% (n=2) Instructs team on protocol registration |
| Team Member (5) | 100% (n=5)Writes final search strategy and translations  100% (n=5) Executes search & exports results  100% (n=5) Writes search methods  60% (n=4) Recommends databases/search environments  60% (n=4) Manages citation/screening software |
